# Gender Differences in Processing Fearful and Angry Body Expressions

**DOI:** 10.3389/fnbeh.2018.00164

**Published:** 2018-07-26

**Authors:** Zhenhong He, Zhenli Liu, Ju Wang, Dandan Zhang

**Affiliations:** ^1^Department of Psychology, College of Psychology and Sociology, Shenzhen University, Shenzhen, China; ^2^Division of Neuroscience and Experimental Psychology, School of Biological Sciences, University of Manchester, Manchester, United Kingdom; ^3^Shenzhen Key Laboratory of Affective and Social Cognitive Science, Shenzhen University, Shenzhen, China

**Keywords:** emotion, body expression, gender difference, affective arousal, event-related potentials

## Abstract

Previous studies have demonstrated differential perception of body expressions between males and females. However, only two recent studies (Kret et al., 2011; Krüger et al., 2013) explored the interaction effect between observer gender and subject gender, and it remains unclear whether this interaction between the two gender factors is gender-congruent (i.e., better recognition of emotions expressed by subjects of the same gender) or gender-incongruent (i.e., better recognition of emotions expressed by subjects of the opposite gender). Here, we used event-related potentials (ERPs) to investigate the recognition of fearful and angry body expressions posed by males and females. Male and female observers also completed an affective rating task (including valence, intensity, and arousal ratings). Behavioral results showed that male observers reported higher arousal rating scores for angry body expressions posed by females than males. ERP data showed that when recognizing angry body expressions, female observers had larger P1 for male than female bodies, while male observers had larger P3 for female than male bodies. These results indicate gender-incongruent effects in early and later stages of body expression processing, which fits well with the evolutionary theory that females mainly play a role in care of offspring while males mainly play a role in family guarding and protection. Furthermore, it is found that in both angry and fearful conditions male observers exhibited a larger N170 for male than female bodies, and female observers showed a larger N170 for female than male bodies. This gender-incongruent effect in the structural encoding stage of processing may be due to the familiarity of the body configural features of the same gender. The current results provide insights into the significant role of gender in body expression processing, helping us understand the issue of gender vulnerability associated with psychiatric disorders characterized by deficits of body language reading.

## Introduction

Successful social interaction requires correctly decoding and recognizing emotional signals from the human body (Krüger et al., 2013; Enea and Iancu, 2016), which is an important medium of emotional communication (De Meijer, 1989; de Gelder, 2009; de Gelder et al., 2010). Compared with the face, the body is considered a more reliable carrier of affective information (Aviezer et al., 2012). When emotions expressed by faces and bodies are incongruent, the emotions conveyed by the body can bias the perception and recognition of facially expressed emotions (Meeren et al., 2005; Van den Stock et al., 2007; Aviezer et al., 2012).

It is noticeable that the processing of emotions expressed by the body is strongly affected by gender differences (Trnka et al., 2007; Aleong and Paus, 2010; Alaerts et al., 2011; Kret et al., 2011; Krüger et al., 2013). For the gender of the observer of body expressions, studies have demonstrated that females are better in recognition of hostile, angry body actions than males, whereas males surpass females in recognition of happy body expressions (Sokolov et al., 2011; Krüger et al., 2013). In addition, Kleinsmith et al. (2006) found shorter response times in female compared to male observers when they recognized emotion from avatar postures, highlighting that the gender of the observer did influence the processing of body expressions. For the gender of the subjects of body expressions, it is believed that males are stereotypically angrier than females, whereas females are more fearful than males (Plant et al., 2000; Kret and de Gelder, 2013; Zibrek et al., 2015). In addition, Johnson et al. (2011) found that subjects with angry body expression were largely judged to be male whereas subjects with sad body expression were more likely to be judged as female, indicating that gender stereotyping affects the decoding of emotional body expressions. However, while previous studies have provided valuable understanding of the influence of gender differences of either observers or subjects of body expressions, the interaction of the two gender factors has only been preliminarily investigated in two studies. Using subtle emotions displayed by point-light locomotion, Krüger et al. (2013) found that male observers had higher recognition accuracy than female observers for happy body expressions portrayed by females, whereas female observers exhibited a tendency to show better performance than male observers for angry body expressions portrayed by males. However, caution should be exercised in generalizing these findings because the gender information conveyed by point-light pictures is very limited (Pollick et al., 2005). Another study by Kret et al. (2011) presented movie clips of fearful and angry body expressions to participants, finding increased activity in brain regions including the extrastriate body area (EBA), superior temporal sulcus, and fusiform gyrus in male observers when they attended to male threatening vs. neutral body expressions. Therefore, the two previous studies of observer and subject gender factors obtained opposite results: Kret et al. (2011) reported a better recognition of emotions expressed by subjects of the same gender, while Krüger et al. (2013) reported a better recognition of emotions expressed by the opposite gender. Furthermore, the two studies used stimuli that contained motion information. This may induce different cognitive processes between male and female observers that are due to motion rather than emotion (e.g., females perform better than males in recognition of non-emotional actions; Alaerts et al., 2011; Sokolov et al., 2011).

To address these issues, the current study used static pictures of body expressions to exclude the potential confounding factor of motion processing (Alaerts et al., 2011; Volkova et al., 2014). In light of previous related studies (Kret et al., 2011; Krüger et al., 2013), it is hoped that this study could further reveal whether an interaction effect of observer and subject gender for body expressions is gender-congruent (i.e., better recognition of emotions expressed by subjects of the same gender) or gender-incongruent (i.e., better recognition of emotions expressed by the opposite gender). In addition to behavioral measures, this study employed event-related potentials (ERPs) to provide neural correlates of gender effects upon body expression processing with high temporal resolution. We were interested in four ERP components that are associated with visual perception and recognition of body expressions. The first is the occipital P1: an early emotional effect on P1 has been revealed for angry and fearful body expressions, demonstrating a rapid detection of threatening body emotions at the earliest stage of visual processing (Meeren et al., 2005; van Heijnsbergen et al., 2007). The second component is the frontal N1, which is associated with early attention allocation (Naatanen, 1988; Hsu et al., 2015). The third component is the occipito-temporal N170, which is generated from the EBA and is an index of early configural encoding of body images and could be modulated by body expressions (Stekelenburg and de Gelder, 2004; Gliga and Dehaene-Lambertz, 2005; Meeren et al., 2005; Righart and de Gelder, 2007; Taylor et al., 2010). The fourth component is the parietal P3: a recent study demonstrated that this component is influenced by top-down attention and affective arousal due to body expressions (Hietanen et al., 2014). Given the evidence that females are more sensitive to threatening information than males (female negativity bias; Lithari et al., 2010; Gardener et al., 2013), and that threatening body expressions performed by males are potentially more harmful (Kret et al., 2011), we hypothesized an interaction of the two gender factors, that is, females would show a heightened sensitivity to threatening body emotion expressed by males. Accordingly, interaction effects also were expected to be shown in the ERP components P1, N1, N170 and P3. However, no detailed hypothesis was formulated with regards to the ERP findings due to the absence of prior studies on this specific topic.

## Materials and Methods

### Participants

Forty undergraduates (20 females) aged 18–26 years participated in this study. All were right-handed and had normal vision (with or without correction). All participants provided written informed consent prior to the experiment. The experimental protocol was approved by the Ethics Committee of Shenzhen University and was in compliance with the 1964 Helsinki Declaration and its later amendments, and this study was performed in strict accordance with the approved guidelines.

### Stimuli

The body stimuli were adapted from a validated stimulus set, i.e., the Bodily Expressive Action Stimulus Test (BEAST; de Gelder and Van den Stock, 2011). A total number of 40 pictures (20 fearful and 20 angry ones) of body expressions were used. The number of actors and actresses was equal in fearful and angry pictures. These two emotions were selected because they are both negative emotions associated with evolutionarily relevant threat situations (Meeren et al., 2005), with fear representing the potential threat and danger in the environment and anger signifying a direct threat and aggression (Marsh et al., 2005; Leppaenen and Nelson, 2012; Borgomaneri et al., 2015). The two stimuli are evolutionarily meaningful and may show a significant level of gender difference. The 40 pictures were selected from BEAST because they had comparable ratings of valence (*t*_(38)_ = 1.1, *p* = 0.26; mean ± standard deviation: anger = 2.9 ± 0.5, fear = 2.7 ± 0.5) and arousal (*t*_(38)_ = −0.9, *p* = 0.38; anger = 5.9 ± 1.3, fear = 6.3 ± 1.0) according to a previous survey (on a 9-point scale) including another 40 Chinese participants. All stimuli were centrally presented in gray scale on the white background with the same contrast and brightness (2.0° × 5°).

### Procedure

**Figure 1:**
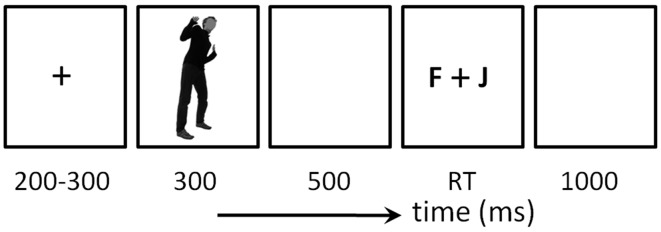
Illustration of one experimental trial in this study.

As shown in Figure 1, stimuli were presented for 300 ms. Participants were required to discriminate, as accurately and rapidly as possible, the body expression category (fear or anger) with the masked face in each trial by pressing the “F” or “J” button on the computer keyboard with their left or right index finger. All body pictures were presented three times in a random order, resulting in 30 trials in each condition. The experiment had a total of 240 trials (30 trials × two emotional types × two gender of observers × two gender of actors/actresses).

After the EEG experiment, participants completed an affective rating task, in which the valence, emotional intensity and arousal of each picture was reported on a 9-point scale.

### Data Recording and Analysis

Brain electrical activity was recorded referentially against left mastoid and off-line re-referenced to average reference, by a 64-channel amplifier using a standard 10-20 system (Brain Products, Gilching, Germany). EEG data were collected with electrode impedances kept below 5 kΩ. Ocular artifacts were removed from EEGs using a regression procedure implemented in NeuroScan software (Scan 4.3).

The data analysis and result display in this study were performed using Matlab R2011a (MathWorks, Natick, MA, USA). The recorded EEG data were filtered (0.01–30 Hz) and segmented beginning 200 ms prior to the onset of stimulus and lasting for 800 ms. All epochs were baseline-corrected with respect to the mean voltage over the 200 ms preceding the onset of stimulus, followed by averaging in association with experimental conditions based on behaviorally correct trials.

We analyzed the amplitudes and latencies of the occipital P1, the frontal N1, the occipito-temporal N170 and the parietal P3 across different sets of electrodes according to the grand-mean ERP topographies and relevant literatures (Stekelenburg and de Gelder, 2004; van Heijnsbergen et al., 2007; Jessen and Kotz, 2011; Gu et al., 2013). The P1 was measured using the peak amplitude and peak latency at electrode sites of O1 and PO3 for the left hemisphere, and at O2 and PO4 for the right hemisphere (time window = 90–130 ms post stimulus). The N1 was measured using the peak amplitude and peak latency at electrode sites of FCz, FC1 and FC2 between 90 ms and 130 ms. The N170 was measured using the peak amplitude and peak latency at electrode sites of P7 and PO7 for the left hemisphere, and at P8 and PO8 for the right hemisphere (time window = 150 ms and 200 ms). The P3 was measured using the average amplitude at electrode sites of Pz, P1 and P2 between 350 ms and 800 ms post stimulus.

### Statistics

Statistical analysis was performed using SPSS Statistics 20.0 (IBM, Somers, NY, USA). Descriptive data were presented as mean ± standard deviation. The significance level was set at 0.05.

Three-way repeated-measures analysis of variance (ANOVA) was performed on measurements of behavioral and ERP data, with emotion (fear and anger) and gender of body expression (male actor and female actress) as the within-subject factors, and gender of observer (male and female subject) as the between-subject factor. For the measurements of P1 and N170 components, four-way repeated-measures ANOVA was performed, adding hemisphere (left and right) as another within-subject factor. Significant interactions were analyzed using simple effects model. Partial eta-squared $\eta_{\text{p}}^{2}$ was reported to demonstrate the effect size.

## Results

For the sake of brevity, this section only reports significant findings.

### Affective Rating Task

For arousal rating, the main effect of emotion was significant (*F*_(1,38)_ = 5.61, *p* = 0.023, $\eta_{\text{p}}^{2}$ = 0.129); fearful expressions (5.0 ± 1.5) were evaluated as more aroused than angry expressions (4.6 ± 1.6). The main effect of gender of body expression was significant (*F*_(1,38)_ = 7.58, *p* = 0.009, $\eta_{\text{p}}^{2}$ = 0.166); body expressions of females (4.9 ± 1.5) were evaluated as more aroused than those of males (4.7 ± 1.5). More importantly, the interaction effect between emotion and gender of observer was significant (*F*_(1,38)_ = 8.51, *p* = 0.006, $\eta_{\text{p}}^{2}$ = 0.183); while female observers (5.6 ± 1.2) rated fearful body expressions more aroused than male observers (4.4 ± 1.5; *F*_(1,38)_ = 8.89, *p* = 0.005), this difference between male and female observers was not observed for angry body expressions (*F* < 1). Furthermore, the interaction effect of emotion × gender of observer × gender of body expression was significant (*F*_(1,38)_ = 4.96, *p* = 0.032, $\eta_{\text{p}}^{2}$ = 0.115). While male observers evaluated female angry expressions (4.8 ± 1.4) more aroused than male angry expressions (4.2 ± 1.5; *F*_(1,38)_ = 16.6, *p* < 0.002), the difference in arousal between male and female expressions was not observed for the other conditions.

**Figure 2:**
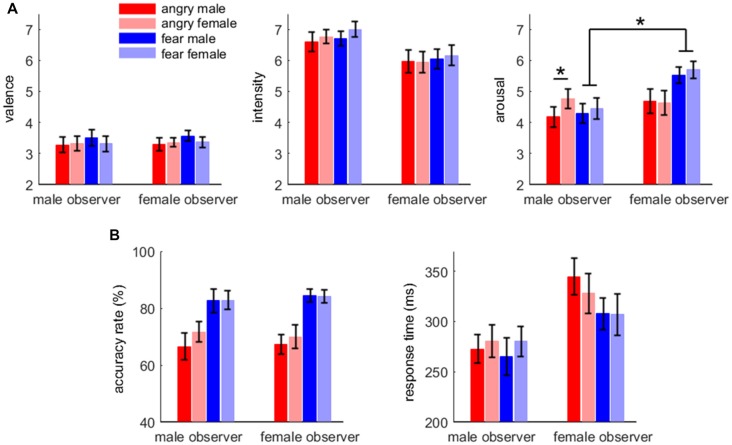
Affective rating **(A)** and behavioral results **(B)**. Bars represent ± standard error of the mean. Significant interaction effect is denoted by an asterisk.

For valence (3.4 ± 1.0) and intensity (6.4 ± 1.4), no significant difference was found across conditions. The affective rating results are shown in Figure 2A.

### Emotion Discrimination Task: Behavioral Data

For accuracy rate, the main effect of emotion was significant (*F*_(1,38)_ = 29.2, *p* < 0.001, $\eta_{\text{p}}^{2}$ = 0.434); the accuracy rate for fearful body expressions (83.5 ± 13.8%) was higher than that for angry body expressions (68.9 ± 17.7%).

For response time, the main effect of gender of observer was significant (*F*_(1,38)_ = 4.65, *p* = 0.037, $\eta_{\text{p}}^{2}$ = 0.109); male observers (275 ± 70 ms) responded more quickly than female observers (322 ± 83 ms). The main effect of emotion was significant (*F*_(1,38)_ = 5.74, *p* = 0.022, $\eta_{\text{p}}^{2}$ = 0.131); the response for fearful body expressions (290 ± 78 ms) was faster than that for angry body expressions (306 ± 81 ms). The behavioral results are shown in Figure 2B.

### Emotion Discrimination Task: ERP Data

#### P1 Component

**Figure 3:**
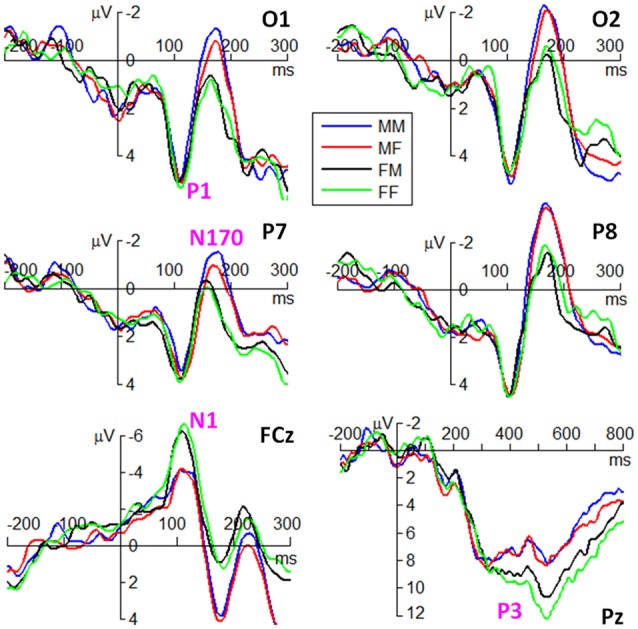
Grand average event-related potentials (ERPs) of the P1, N1, N170 and P3 components at the indicated electrode sites in fear condition. M represents male. F represents female. The first latter represents gender of observers. The second latter represents gender of body expressions.

**Figure 4:**
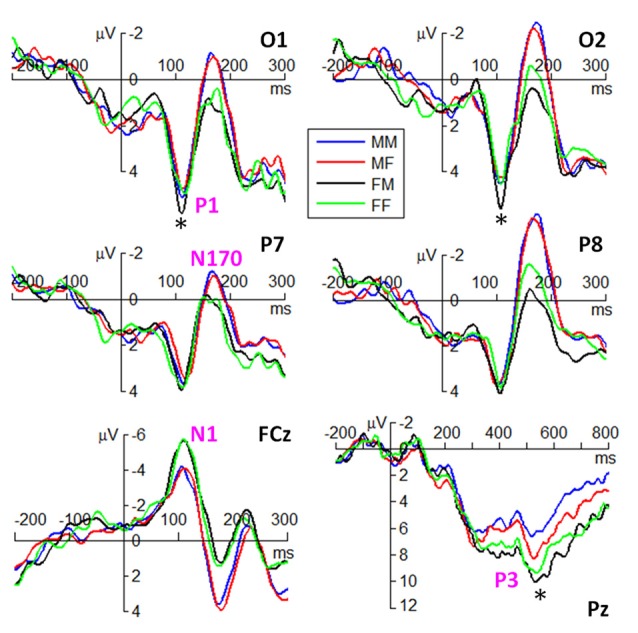
Grand average ERPs of the P1, N1, N170 and P3 components at the indicated electrode sites in anger condition. The symbol * denotes a significant level of *p* < 0.05.

ERP waveforms of the four components are shown in Figures 3, 4 in fear and anger conditions respectively. For peak amplitude, the interaction effect of emotion × gender of observer × gender of body expression was marginally significant (*F*_(1,38)_ = 4.09, *p* = 0.050, $\eta_{\text{p}}^{2}$ = 0.097). While larger P1 was evoked by angry body expressions postured by males (6.54 ± 3.55 μV) than females (5.54 ± 3.21 μV) in female observers (*F*_(1,38)_ = 9.12, *p* = 0.004), this amplitude difference between male and female body expressions was not observed for the other conditions.

For peak latency, the main effect of emotion was significant (*F*_(1,38)_ = 12.2, *p* = 0.001, $\eta_{\text{p}}^{2}$ = 0.244); the P1 evoked by fearful body expressions (112 ± 10 ms) peaked earlier than that evoked by angry body expressions (116 ± 9 ms). The main effect of hemisphere was significant (*F*_(1,38)_ = 5.28, *p* = 0.027, $\eta_{\text{p}}^{2}$ = 0.122); the P1 latency in the right hemisphere (112 ± 10 ms) was shorter than that in the left hemisphere (115 ± 10 ms).

#### N1 Component

For peak amplitude, the main effect of gender of observer was significant (*F*_(1,38)_ = 4.45, *p* = 0.041, $\eta_{\text{p}}^{2}$ = 0.105); the amplitudes in female observers (−7.26 ± 3.95 μV) were larger than that in male observers (−5.01 ± 3.27 μV).

For peak latency, the main effect of emotion was significant (*F*_(1,38)_ = 4.19, *p* = 0.048, $\eta_{\text{p}}^{2}$ = 0.099); the N1 evoked by angry body expressions (111 ± 13 ms) peaked earlier than that evoked by fearful body expressions (113 ± 13 ms).

#### N170 Component

For peak amplitude, the main effect of gender of observer was marginally significant (*F*_(1,38)_ = 4.09, *p* = 0.050, $\eta_{\text{p}}^{2}$ = 0.097); the N170 evoked in male observers (−3.42 ± 3.37 μV) were larger than that evoked in female observers (−1.86 ± 3.11 μV). The main effect of hemisphere was significant (*F*_(1,38)_ = 12.9, *p* = 0.001, $\eta_{\text{p}}^{2}$ = 0.253); the N170 was larger in the right (−3.62 ± 3.68 μV) than in the left hemisphere (−1.66 ± 2.60 μV). More importantly, the interaction effect between gender of observer and gender of body expression was significant (*F*_(1,38)_ = 7.46, *p* = 0.010, $\eta_{\text{p}}^{2}$ = 0.164); while male body expressions (−3.56 ± 2.60 μV) evoked larger N170 than female body expressions (−3.27 ± 2.60 μV) in male observers (*F*_(1,38)_ = 5.01, *p* = 0.031), female body expressions (−1.96 ± 2.60 μV) evoked slightly larger N170 than male body expressions (−1.75 ± 2.60 μV) in female observers (*F*_(1,38)_ = 2.64, *p* = 0.112). Furthermore, the interaction effect of emotion by gender of observer by hemisphere was significant (*F*_(1,38)_ = 4.16, *p* = 0.048, $\eta_{\text{p}}^{2}$ = 0.099). Angry body expressions evoked larger N170 in male observers (−4.60 ± 3.27 μV) than female observers (−2.28 ± 3.67 μV) in the right hemisphere (*F*_(1,38)_ = 4.63, *p* = 0.038); this amplitude difference between male and female observers was not observed for the other conditions.

In addition, the interaction effect between emotion and gender of body expression was significant (*F*_(1,38)_ = 5.70, *p* = 0.022, $\eta_{\text{p}}^{2}$ = 0.131); while the N170 evoked by male expressions (−2.95 ± 3.47 μV) were larger than that evoked by female expressions (−2.60 ± 3.38 μV) in fear condition (*F*_(1,38)_ = 5.85, *p* = 0.021), this amplitude difference was not significant in anger condition (*F*_(1,38)_ = 1.15, *p* = 0.291). The interaction effect between gender of body expression × hemisphere was significant (*F*_(1,38)_ = 7.52, *p* = 0.009, $\eta_{\text{p}}^{2}$ = 0.165); while male body expressions (−1.82 ± 2.62 μV) evoked larger N170 than female body expressions (−1.50 ± 2.59 μV) in the left hemisphere (*F*_(1,38)_ = 7.83, *p* = 0.008), this effect was not observed for the right hemisphere (*F*_(1,38)_ = 2.27, *p* = 0.140).

For peak latency, no significant difference was found across conditions.

#### P3 Component

For average amplitude, the main effect of emotion was significant (*F*_(1,38)_ = 10.1, *p* = 0.003, $\eta_{\text{p}}^{2}$ = 0.210); fearful expressions (7.41 ± 3.84 μV) evoked larger P3 compared to angry expressions (6.31 ± 3.64 μV). The main effect of gender of observer was significant (*F*_(1,38)_ = 5.77, *p* = 0.021, $\eta_{\text{p}}^{2}$ = 0.132); the P3 elicited in female observers (8.08 ± 3.73 μV) was larger than that elicited in male observers (5.65 ± 3.4 μV). More importantly, the interaction effect of emotion × gender of observer × gender of body expression was significant (*F*_(1,38)_ = 4.73, *p* = 0.036, $\eta_{\text{p}}^{2}$ = 0.111). While larger P3 was evoked by angry body expressions postured by females (5.63 ± 2.99 μV) than males (4.58 ± 2.52 μV) in male observers (*F*_(1,38)_ = 4.35, *p* = 0.044), this amplitude difference between male and female body expressions was not observed for the other conditions.

## Discussion

The findings of the current study suggest that the recognition and conveyance of fearful and angry body expressions is strongly affected by gender. The most distinct finding is the gender-incongruent effect observed in P1 and P3 amplitudes. That is, female observers had larger P1 for male than female angry expressions, while male observers had larger P3 for female than male angry expressions. In light of the cognitive processes associated with the P1 component in body expression processing (Meeren et al., 2005; van Heijnsbergen et al., 2007), the current results suggest that at an early stage of visual processing, female observers have stronger vigilance for angry male than angry female bodies. This vigilance occurs at the earliest stage of visual processing, even before configural processing of the human body (indexed by the N170 component; Stekelenburg and de Gelder, 2004; Meeren et al., 2005; Righart and de Gelder, 2007). This early effect (P1) is evolutionarily adaptive to females, although it did not result in better performance in female vs. male observers for angry male body identification, as revealed in Krüger et al.’s (2013) study. Bearing in mind that from the evolutionary point of view the role of the female is often related to offspring caring, females should be sensitive to aggressive or threatening cues including angry body expressions so as to help keep their offspring away from danger (Krüger et al., 2013). Since the anger posed by males is more frequent (Bosson et al., 2009), more threatening and more physically harmful than that posed by females (Lassek and Gaulin, 2009; Kret et al., 2011; Kret and de Gelder, 2012; Tay, 2015), it could be more rapidly detected and processed by females. Another explanation is that females usually have smaller body size and less strength compared to males, so females should detect and react to the anger expressed by males as early as possible to keep themselves safe. In accordance with these ideas, behavioral evidence suggests that females respond faster to male compared to female angry facial expressions (Rotteveel and Phaf, 2004). For males, quickly detecting angry signals from other males is also important because males are often engaged in male-male aggressive behavior associated with resource supply and mate-seeking performance (Kret and de Gelder, 2012; Tay, 2015). However, we did not observe such effects in the P1 of male observers. This may be because females usually show greater cortical activation at earlier stages in visual processing of biological motion compared to males (Pavlova et al., 2015). However, further experimental evidence is required to evaluate this explanation.

Another important interaction effect was found in later stages of processing. Larger P3 amplitudes were evoked in male observers when they watched angry body expressions posed by female relative to male subjects. The P3 in previous studies has been found to be larger for high-arousal compared to neutral human body expressions (Hietanen et al., 2014). In accordance with this study, the P3 effect likely reflects a high affective arousal in male observers in response to angry female bodies, as revealed by the behavioral arousal rating. Adopting a similar time window for P3 analysis (200–650 ms), previous studies show that males but not females exhibited larger late slow waves to attractive female faces than male faces, which could also be explained by arousal (van Hooff et al., 2011). This explanation is in line with a neuroimaging study that demonstrated larger amygdala activation was found in males when observing female compared to male faces (Fischer et al., 2004). Since the amygdala is also a critical region in response to body affective information (Peelen et al., 2007), the current finding regarding P3 could be due to this amygdala effect when emotional cues such as faces and bodies are presented. According to evolutionary based hypotheses, to maintain power and privilege, males should utilize a variety of ways to coerce and control female partners (Dobash and Dobash, 1979). It is suggested that high sensitivity and arousal to anger from female partners may help males to detect potential threats to their power and privilege. The lack of similar gender-differential P3 effects in female observers is consistent with the insignificant behavioral arousal rating, indicating that affective arousal in females evoked by threatening expressions was not modulated by body gender. This may be because females allocate relatively fewer cognitive resources at later stages of body stimuli processing, as revealed by a recent magnetoencephalography study (Pavlova et al., 2015).

In the present study, the above gender differential effects in processing body expressions were found in only the anger condition. Although fear and anger both constitute threatening signals, anger is a more interactive signal because coping with another’s anger involves additional socially adaptive behaviors relative to coping with another’s fear (Pichon et al., 2009). Thus, the processing of fearful and angry body expressions entails both common but also specific neural representations: whereas fear mainly triggers activity in the right temporoparietal junction, anger elicits activation in the temporal lobe, premotor cortex and ventromedial prefrontal cortex (Pichon et al., 2009). Notably, temporal lobe and premotor cortex have been reported to be the gender-specific regions of human body processing in several previous studies (Kret et al., 2011; Kana and Travers, 2012; Pavlova, 2017). These regional differences may provide the explanation for why the observed gender effects are much stronger in the anger than in the fear condition.

Unlike the findings for the P1 and P3 components, this study revealed a gender-congruent effect in the structural encoding stage indexed by N170. In particular, males showed larger N170 for male than female bodies and females showed a tendency to have larger N170 amplitudes for female than male bodies. This result suggests that observers are more sensitive to the configural information of body expressions posed by subjects of the same gender than the opposite gender. Previous studies have indicated that familiar body stimuli could evoke more prominent configural processing than unfamiliar stimuli (Reed et al., 2003), which explains why female dancers performed better than male dancers in discrimination of point-light dancing movements expressed by females (Calvo-Merino et al., 2010). In line with this idea, the current N170 result indicates that the configural perception of body expressions is enhanced by familiarity of body expressions posed by subjects of the same gender as the observer.

This study also discovered that female observers showed larger N1 and P3 amplitudes than male observers in response to both threatening body expressions (anger and fear). The N1 result replicated a recent finding showing that females displayed a larger N1 in response to negative images than males (Lithari et al., 2010; Gardener et al., 2013). This N1 finding indicates that females are more sensitive to threatening information than males in early stages of emotional processing and reactivity. The P3 result is consistent with previous studies showing that relative to males, females had an enhanced P3 for negative pictures and expressions of suffering in infants (Proverbio et al., 2006; Li et al., 2008). The enhanced P3 in female observers indicates that females allocate more cognitive resources than males at the stage of in-depth evaluation of negative emotions (Gard and Kring, 2007; Gardener et al., 2013).

There are a number of limitations of this study. First, the current work only explored gender differences in young adult participants. Previous studies have demonstrated that when processing point-light body movements, gender differences in brain activation (e.g., temporal lobe and amygdala) are less significant in 4-to-16-year-old children and adolescents as compared to adults (Anderson et al., 2013). Therefore, generalization of the current findings for all age groups (e.g., adolescents and elderly people) should be treated with caution. Second, we did not measure hormonal information and thus we cannot rule out the possibility that the observed gender effects might be partly driven by gender dimorphic endocrine status. In particular, females usually show hormonal fluctuations across the menstrual cycle, which may lead to fluctuations of behavioral performance in different tasks (Hampson and Kimura, 1988). For example, previous studies have shown that females showed differential performance in the identification of emotional faces, depending on the phase of the menstrual cycle (Guapo et al., 2009). Third, due to the low spatial resolution of electroencephalogram (EEG), the present study could not provide information on the specific brain regions accounting for the observed gender differences. There is much evidence indicating that the neural substrates underlying emotional body language reading are gender specific, i.e., the EBA, superior temporal sulcus, anterior insula, superior parietal lobule, pre-supplementary motor area and premotor cortex are reported to mediate gender differences in processing body expressions (Kret et al., 2011; Kana and Travers, 2012). Future studies should explore the time course and functional anatomy of gender differences, for example by using simultaneous EEG-functional magnetic resonance imaging.

Our results have potential clinical relevance. Most psychiatric disorders characterized by social cognition deficits (e.g., autism and depression) have gender-specific patterns of prevalence and severity. For example, males have a higher risk than females of suffering from autism spectrum disorder (Newschaffer et al., 2007), while females are more affected by depression and anxiety disorders. Clarifying the gender impact could help provide novel insights into understanding of gender vulnerability in these psychiatric disorders (Craske, 2003).

In conclusion, the present study discovered distinct interaction effects between the gender of observers and the gender of subjects of body expressions. At the early (P1) and late (P3) processing stages, we discovered gender-incongruent effects of body expression processing. However, gender-congruent effects were exhibited in the structural encoding stage indexed by N170, which may be due to the familiarity of the configural features of bodies of the same gender. Taken together, the current results highlight the significance of gender as a modulating factor in the processing of body expressions. Further studies focusing on the gender dimorphism issue are needed to facilitate the understanding of differential gender vulnerability in psychiatric disorders characterized by social cognition deficits including impairment of body language reading (Pavlova, 2012, 2017).

## Author Contributions

DZ designed the study. ZL conducted the experiment. ZH and DZ analyzed the data. ZH, ZL, JW and DZ contributed to the manuscript.

## Conflict of Interest Statement

The authors declare that the research was conducted in the absence of any commercial or financial relationships that could be construed as a potential conflict of interest.
